# Skipping breakfast is associated with adiposity markers especially when sleep time is adequate in adolescents

**DOI:** 10.1038/s41598-019-42859-7

**Published:** 2019-04-23

**Authors:** Elsie C. O. Forkert, Augusto Cesar Ferreira De Moraes, Heráclito Barbosa Carvalho, Yannis Manios, Kurt Widhalm, Marcela González-Gross, Angel Gutierrez, Anthony Kafatos, Laura Censi, Stefaan De Henauw, Luis A. Moreno

**Affiliations:** 1 YCARE (Youth/Child cArdiovascular Risk and Evironmental) Research Group, Departamento de Medicina Preventiva, Faculdade de Medicina FMUSP, Universidade de Sao Paulo, Sao Paulo, SP Brazil; 2 GENUD (Growth, Exercise, NUtrition and Development) Research Group, Facultad de Ciencias de la Salud de la Universidad de Zaragoza,, Instituto Agroalimentário de Aragón (IA2), Zaragoza, Spain; 3 Department of Preventive Medicine - Visiting Professor, School of Medicine, University of São Paulo, Sao Paulo, Brazil; 4 Department of Nutrition and Dietetics, Harokopio University, Athens, Greece; 5 Department of Pediatrics, Paracelsus Medical University, Salzburg, Austria; 6 ImFINE Research Group. Department of Health and Human Performance, Faculty of Physical Activity and Sport-INEF, Technical University of Madrid, and Centro de Investigación Biomédica en Red Fisiopatología de la Obesidad y Nutrición(CIBEROBN), Madrid, Spain; 7 Institut für Ernährungs-und Lebensmittelwissenschaften–Humanernährung, Rheinische Friedrich-Wilhelms, Universität Bonn, Bonn, Germany; 8 Department of Medical Physiology, School of Medicine, University of Granada, Granada, Spain; 9 Preventive Medicine and Nutrition Unit, University of Crete School of Medicine, Heraklion, Crete, Greece; 10 Council for Agricultural Research and Economics-Research Center for Food and Nutrition, Rome, Italy; 11 Department of Public Health, Ghent University, Ghent, Belgium; 12 Insttituto Agroalimentario de Aragón (IA2), Instituto Investigación Sanitaria Aragón (IIS Aragón) and Centro de Investigación Biomédica en Red Fisiopatología de la Obesidad y Nutrición (CIBEROBN), Zaragoza, Spain

**Keywords:** Health care, Risk factors

## Abstract

Adolescence is a critical stage of development and has an important influence on energy balance-related behaviours (EBRBs). When adolescents are associated with obesity it can lead to increased cardiometabolic risk. Here we assess if EBRBs adopted by adolescents included in a subsample are associated with markers of total and abdominal adiposity in a multicentre European study, *Healthy Lifestyle in Europe by Nutrition in Adolescence* (HELENA-CSS) and a Brazilian study, *Brazilian Cardiovascular Adolescent Health* (BRACAH study), and whether sleep duration influence the association between skipping breakfast, physical activity and sedentary behaviours, with total and abdominal obesity (AO). Multilevel linear regression models using fixed and random intercepts were used to analyse the association between markers of obesity and EBRBs. Skipping breakfast was the prevalent behaviour in association with obesity among European and Brazilian boys besides European girls, even after stratification by sleep time. Moreover, European boys who slept properly and skipped breakfast had an increased waist circumference (WC), while body mass index (BMI) increased in Brazilian boys. Among Brazilian boys less sleep was protective for total obesity (β = −0.93 kg/m^2^; 95% CI: −1.80; −0.07). European girls when they were more sedentary, showed an increase in WC, especially for those who reported they slept adequately. Skipping breakfast was associated with total and AO in adolescents independent of sleep duration.

## Introduction

Overweight/obesity represents a growing problem because they seriously affect health, leading to impaired quality of life for both, children and adults^1^. The worldwide prevalence of childhood overweight and obesity rose from 4.2% to 6.7% between 1990 to 2010. In this way, the expected trend is to reach 60 million (9.1%) by 2020^2^. Regarding abdominal obesity (AO) it has been identified as atherosclerosis’ determinant in young adults, independent of traditional risk factors [e.g. BMI, adiponectin levels, and markers of insulin resistance (IR)]^3^. Furthermore, in children and adolescents it is associated with cardiometabolic risk factors, such as IR, leading to the development of *type-*2 diabetes^4^. Behaviours are the result of learning history through biological (age, sex, genetic and psychological factors) and environmental influences, which in turn will influence the energy balance^5^. Identified as important in addressing childhood obesity prevention and health promotion are the several energy balance-related behaviours (EBRBs). According to Bandelli *et al*.^6^, EBRBs are behaviours which handled on energy-balance and will yield weight gain or loss, amongst their physical activity, sedentary, dietary behaviors^6^ and sleep habits^7^ of special relevance among schoolchildren^8^. Once EBRBs are associated with adiposity and this is influenced by the environment and biological factors, these behaviours should be examined within a global strategy^5^. Though the majority of the literature has focused on each behaviour separately, analysing the co-occurrence of several EBRBs could help identify subgroups at an increased risk of developing obesity^9^. In a multinational study the International Study of Childhood Obesity, Lifestyle and the Environment(ISCOLE), healthier eating patterns are assumed by children when they attend to more movement behaviours, according to the recommendations^10^. Combinations of behaviours as a set of several EBRBs are more prevalent than expected based on the prevalence of each behaviour separately^11^. Adolescence as a critical period for the acquisition of healthy behaviours, healthy behaviours should be encouraged and well absorbed to prevent (or postpone) the onset of non-communicable diseases^12^. Among adolescents, scientists point out the role of behavioural factors in the aetiology of obesity, especially dietary habits, physical activity (PA) and sedentary behaviours (SB)^13^. However, other EBRBs have been identified as important, such as inadequate sleep time^14^ and skipping breakfast^15^. Sleep restriction through different mechanisms plays an important role in adiposity, since it may influence the healthy-lifestyle (increasing SB and decreasing PA)^16^. In the past decades, there was an accelerated growth on skipping breakfast at least among US children and adolescents, especially in older adolescents, mainly associated with behavioural changes^17^, which raises important points about this behaviour in association with obesity.

In this context, we addressed two hypotheses: **(i)** the EBRBs adopted by adolescents are associated with markers of total and abdominal obesity of a multicentre European study and a Brazilian study, and (**ii**) the sleep time may influence the association between skipping breakfast, physical activity and sedentary behaviours, with total and abdominal obesity.

## Methods

### Study design and sampling

The current study includes data from two school-based cross-sectional studies, one European and the other Brazilian. In both studies, male and female adolescents from private and public schools in urban areas were eligible. The European study, *Healthy Lifestyle in Europe by Nutrition in Adolescence Cross-Sectional Study* (HELENA-CSS), which was carried out from 2006 to 2007, is a multi-centre study performed in 3,528 adolescents (52.3% girls; aged 12.5–17.5 years), stratified by age, sex, region and socio-economic status. In ten cities from nine European countries: Athens(Greece), Dortmund (Germany), Ghent (Belgium), Heraklion(Greece), Lille (France), Pecs (Hungary), Rome (Italy), Stockholm (Sweden), Vienna (Austria), Zaragoza(Spain), their nutritional status and lifestyle behaviour were analysed. The methodological aspects and standardisation, harmonisation and analysis strategies were described thoroughly elsewhere^18^. Participants who met the following criteria were included: correct age range, not participating simultaneously in another clinical trial and being free of any acute infection in the previous week. From the total sample, this analysis included 2,371 adolescents (54.8% girls) having available data from the used variables (see Supplementary File sample size).

Parents and/or legal guardian and adolescents signed the written consent form attesting the participation of their children in the study, after receiving complete information about it. The study was approved by the Research Ethics Committee of the centres involved (for most, this was the countries’ Minister of Health), and was accomplished following the ethical principles of the Declaration of Helsinki, 1964 (revision of Edinburgh, Scotland 2000)^19^.

The Brazilian study, *Brazilian Cardiovascular Adolescent Health* (BRACAH study) data were collected in 2007, in a city of southern region of Brazil –*Maringá (PR)* with a population of about 330,000 inhabitants. After random selection, a total of 991 adolescents (54.5% girls, aged 14–18years) were included and evaluated regarding to cardiovascular risk factors and health-related behaviours. The complete methodology and sample size of this study has been described elsewhere^20^. Adolescents with orthopaedic problems preventing anthropometric assessments, pregnancy or no parental consent met the exclusion criteria. Parental informed consent was obtained and the adolescents agreed to participate in the study through their assent. The informed consent was obtained from all participants (over 18 years old) or, if participants were under 18, a parent and/or legal guardian attested the participation of their children. The Ethics Committee on Research Involving Human Participants of the University Centre of *Maringá* approved the study, which was authorised by the Ethics Committee on Research Projects of the University of São Paulo in accordance with Brazilian laws.

For the present study, adolescents from HELENA-CSS and BRACAH study were included since they had complete data on age, sex, weight, height and WC. Mother education level and EBRBs as: PA, SB, sleep time and breakfast consumption were also the variables used in this study, which are described in detail below.

## Outcomes

### Anthropometric measurements

*Weight* was measured with an electronic scale (*Type SECA861*) in underwear and without shoes. For *height*, a telescopic height-measuring instrument (*Type SECA225*) was used to the nearest 0.1 cm for barefoot measurement in the Frankfort plane. The *body mass index* (BMI) was calculated through body weight (kg) divided by height (m) squared^21^. Obesity (including overweight) was defined using the international BMI cut-offs suggested by Cole *et al*.^22^, which are based on the populations of several countries. They provide age and sex-specific, centile curves that at age 18 years passed through cut-off points of BMI for adult overweight and obesity as 25 and 30 kg/m^2^, respectively^22^. Abdominal obesity was defined using *waist circumference* (WC) and *waist-to-height ratio* (WHtR). WC was measured in both studies using a non-elastic tape (*TypeSECA200*) to the nearest 0.1 cm in the midpoint between the lowest border of the rib cage and the upper border of the iliac crest. Adolescents were in a standing position, abdomen relaxed and at the end of a normal expiration^21^. WHtR was calculated as WC divided by height, both in centimetres (cm) and was categorised as the presence or absence of abdominal obesity when WHtR ≥ 0.5 or WHtR < 0.5, respectively^23^.

## Independent Variables

*Energy balance–related behaviours* were measured and evaluated through a self-reported questionnaire in both studies.

*Physical activity* (PA) moderate–to-vigorous physical activity (MVPA) levels were assessed using the *International Physical Activity Questionnaire for Adolescents (*IPAQ-A), which was adapted and validated to adolescents from the long version of IPAQ^24^. The IPAQ-A examined PA in different domains such as school (physical education classes and breaks), transport, home or garden and leisure time. The questionnaire was validated with the accelerometers in the adolescents from the HELENA-CSS (*R*^*2*^ = 0.81), and in the BRACAH study this questionnaire showed an excellent agreement and reproducibility (*kappa-values* = 0.91), when physical activity of moderate-to-vigorous was < 300 min/week, following physical activity recommendations for school-age youth^25,26^. In the present study the variable was categorised considering moderate-to-vigorous PA as: ≥ 60 min/day (physically active) or < 60 min/day (insufficiently active).

*Sedentary behaviours* (SB) were examined over a structured questionnaire, which requested habitual screen-time spent in front of television, computer and/or playing video games. “*During weekdays, how many hours do you usually spend watching television*?”;“*During weekdays, how many hours do you usually spend on computers*?”; “*During weekdays, how many hours do you usually spend playing video games*?”. The same questions about weekend days were also required. Total SB time was calculated by summing hours in the week and weekend days and classified according to: 0–2 h/d; >2–4 h/d; ≥4 h/d^27^. The questionnaire was translated and adapted to each participating city, and its reproducibility and reliability were tested: *k-values* = 0.81 in the HELENA-CSS^28^ and *k-values* = 0.79 in the BRACAH study. In the current study this variable was dichotomised as ≤2 hours/day (low) or >2 hours/day (high).

*Skipping breakfast*: The “*Food Choices and Preferences*”(FCP) questionnaire^29^ explored attitudes and concern issues regarding food choices, preferences, healthy eating and lifestyle among adolescents, and also it was used to assess breakfast consumption. Based on the agreement with the statement: “*I often skip breakfast”* participants answered with 7 categories varying from *strongly disagree (1)* to *strongly agree (7)* being the category 4 placed as neither agree nor disagree, categorising the variables breakfast non-skipper (1to3) and breakfast skipper (5to7)^30^. In the BRACAH study consumption of breakfast as well as the other main meals was evaluated by having them whether at home or not^31^. So, the variable was also categorised as not skipping breakfast and skipping breakfast.

*Sleep time* was estimated through habitual sleep duration by questions: “*During weekdays how many hours (or minutes) do you usually sleep?”* and *“During weekend days how many hours (or minutes) do you usually sleep?”* A subsample of 183 adolescents (13–18 years old) answered the questionnaire twice, one week apart to evaluate the reliability and reproducibility of these questions. Excellent agreement showed by the *Cohen’s weighted kappa* of 0.81 during weekdays and 0.96 during weekend days^32,33^. In the final HELENA study sample this subsample did not participate, but also did not differ in age, ethnicity or socioeconomic status. In the present study sleep time was dichotomised as ≥8 hours/day (adequate) or <8 hours/day (inadequate)^34^.

### Potential Confounders

Confounders were analysed:

*Age (years): calculated from birthday and medical examination day

*Centre (cities participants only in the HELENA-CSS)

*Maternal education level: a self-reported questionnaire on the socioeconomic status was applied and assessed information on education level (used as a socioeconomic marker). It encloses four categories: elementary education, lower secondary education, higher secondary education and university degree^35^.

### Statistical analysis

Descriptive analyses were presented as means (quantitative variables) and percentages (qualitative variables) and 95% confidence intervals.

Multilevel linear regression models using fixed and random intercepts (school as contextual factors) stratified by sleep time, sex and study were used to investigate associations between EBRBs (independent variables) with total and abdominal obesity (outcomes). Age, maternal education level and centre entered as confounders. The magnitude of these associations was expressed in unadjusted and adjusted β-coefficients and their specific 95%CI.

In order to clarify the distal and proximal determinants related to the outcomes, in the adjusted analyses, a conceptual framework has been created to show the hierarchical relationship between these determinants, positioned at a contextual and individual level. Centre (only HELENA-CSS) and school were entered as contextual variables (distal exposure). Regarding the individual level, socio-demographic factors such as age, socioeconomic status (mother education level) were the intermediate exposures, and EBRBs to the adolescents constituted the proximal^36^ (see Supplementary Conceptual Framework).

In the unadjusted analysis, each independent and confounder variable was introduced separately. A potential confounder was retained in the multivariate model, when significance level in the unadjusted analysis was *p-values* ≤ 0.20 or any change of 10% in the β-coefficient. In multivariate analysis that followed the hierarchical conceptual model, according to the levels mentioned above, the significance was set at *p-values* < 0.05 *(two-sided)*. All the statistical analyses were stratified by sex and performed by using Stata12 (*Stata Corp., College Station, TX, USA*).

## Results

### Descriptive characteristics

Table 1 presents the main characteristics of adolescents studied. Boys were heavier and taller than girls in both studies and had a larger WC. Otherwise, considering another visceral fat marker, Brazilian girls presented a higher WHtR than other adolescents. Related to EBRBs, European girls had a lower prevalence of sedentary behaviours (>2 h/day), but in counterpart they were less physically active than European boys, although more active (PA ≥ 60 min/day) than Brazilian adolescents. What concerns Brazilian adolescents, girls were more sedentary (>2 h/day) than boys. Regarding breakfast consumption and sleep time, European and Brazilian boys had a lower prevalence of skipping breakfast and adequate sleep time (≥8 h/day) than girls. The association between EBRBs (independent variables) and outcomes (total and abdominal obesity) without adjustments (unadjusted analysis) are presented in Supplementary Table S1. There was a positive association between skipping breakfast and SB with BMI and AO (WC and WHtR) among European girls. In European and Brazilian boys skip breakfast and sleep less than 8 h/day, was respectively associated with increased/decreased of all adiposity indicators.

**Table 1:** Main characteristic of the studied adolescents from HELENA-CSS and BRACAH studies.

	Girls				Boys			
	n	HELENA	n	BRACAH	n	HELENA	n	BRACAH
		mean or % (95%CI)		mean or % (95%CI)		mean or % (95%CI)		mean or % (95%CI)
Age (years)	1372	14.7 (14.6–14.8)	540	16.2 (16.2–16.3)	1154	14.8 (14.7–14.8)	451	16.4 (16.3–16.5)
Weight (kg)	1372	56.3 (55.8–56.9)	540	56.3 (55.5–57.1)	1154	62.5 (61.7–63.3)	451	67.2 (66.0–68.5)
Height (cm)	1372	162.3 (162.0–162.7)	540	162.7 (162.2–163.2)	1154	170.2 (169.7–170.8)	451	175.2 (174.6–175.8)
BMI (kg/m²)	1372	21.3 (21.2–21.5)	540	21.3 (21.0–21.6)	1154	21.4 (21.2–21.7)	451	21.8 (21.5–22.2)
Waist circumference (cm)	1347	70.4 (70.0–70.8)	540	77.3 (76.5–78.1)	1134	74.2 (73.7–74.8)	451	80.4 (79.5–81.4)
Waist to height	1347	0.35(0.34–0.35)	540	0.48 (0.47–0.48)	1134	0.36 (0.36–0.37)	451	0.46 (0.45–0.46)
**Sleep time**	1350		390		1121		331	
≥8 hours/day		66.2 (63.7–68.7)		51.3 (46.0–56.0)		67.2 (64.4–70.0)		57.1 (52.0–62.0)
<8 hours/day		33.8 (31.3–36.3)		48.7 (44.0–54.0)		32.8 (30.1–35.6)		42.9 (38.0–48.0)
**Skip breakfast**	1372		540		1154		451	
no		55.5 (52.8–58.1)		62.2 (58.0–66.0)		64.1 (61.4–67.0)		65.4 (61.0–70.0)
yes		44.5 (42.0–47.2)		37.8 (34.0–42.0)		35.9 (33.1–38.6)		34.6 (30.0–39.0)
**Sedentary behaviour**	1372		540		1154		451	
≤2 hours/day		42.4 (39.8–45.0)		3.0 (2.0–4.0)		21.7 (19.3–24.0)		5.3 (3.0–7.0)
>2 hours/day		57.6 (55.0–60.2)		97.0 (96.0–98.0)		78.3 (76.0–80.7)		94.7 (93.0–97.0)
**Physical activity**	1372		540		1154		451	
≥60 min /day		83.9 (82.0–85.8)		30.6 (27.0–34.0)		85.3 (83.2–87.3)		31.7 (27.0–36.0)
<60 min/day		16.1 (14.2–18.1)		69.4 (66.0–73.0)		14.7 (12.7–16.8)		68.3 (64.0–73.0)
**Education mother**	1322		533		1091		449	
Low education		7.3 (5.9–8.7)		8.4 (6.1–10.8)		7.6 (6.0–9.2)		6.5 (4.2–8.7)
Secondary low education		24.1 (21.7–26.4)		24.8 (21.1–28.4)		25.6 (23.0–28.2)		25.2 (21.1–29.2)
Secondary high education		33.4 (30.8–36.0)		42.8 (38.6–47.0)		31.3 (28.6–34.1)		42.8 (38.2–47.4)
University degree		35.3 (32.7–38.0)		24.0 (20.4–27.7)		35.5 (32.6–38.3)		25.6 (21.6–29.7)

HELENA-CSS: Healthy Lifestyle in Europe by Nutrition in Adolescence cross-sectional study, BRACAH study: Brazilian Cardiovascular Adolescent Health study, 95%CI: confidence interval.

### Individual associations EBRBs x adiposity

After adjusting for potential confounders the significant association of skipping breakfast and SB with adiposity indicators was maintained in European girls. In boys from both studies, skip breakfast was the prevailing behaviour, showing a positive association with the indicators of obesity (BMI, WC and WHtR), being highest with WC and among Brazilian boys who slept less than 8 h/day remained a negative association with BMI.

On other hand, physical activity did not present any association with obesity, even after adjustments by the confounders (Table 2).

**Table 2:** Adjusted* association between EBRBs and general and abdominal obesity, among adolescents studied from HELENA-CSS and BRACAH study.

	n	HELENA	p	n	BRACAH	P
		coef β (95%CI)			coef β (95%CI)	
	**Girls**					
**BMI**						
Sleep time	1301	0.20 (−0.21; 0.61)	0.337	385	0.02 (−0.63; 0.68)	0.941
Skip breakfast	1322	**0.70 (0.32; 1.08)**	**<0.001**	533	−0.10 (−0.69; 0.49)	0.743
Sedentary behaviour	1322	0.30 (−0.09; 0.69)	0.129			
Physical activity	1322	−0.11 (−0.63; 0.40)	0.666	540	0.33 (−0.33; 0.98)	0.330
**Waist circumference**						
Sleep time	1325	−0.02 (−0.92; 0.88)	0.97	385	−0.57 (−2.49; 1.35)	0.560
Skip breakfast	1300	**0.98 (0.12; 1.83)**	**0.025**	533	0.86 (−0.76; 2.47)	0.298
Sedentary behaviour	1300	**1.19 (0.32; 2.06)**	**0.007**			
Physical activity	1347	−0.05(−1.19; 1.08)	0.93	540	−0.03 (−1.83; 1.78)	0.978
**Waist to heigh**t						
**Sleep time**						
Skip breakfast	1300	**0.01 (0.00; 0.01)**	**0.001**			
Sedentary behaviour	1300	**0.01 (0.00; 0.02)**	**<0.001**			
**Physical activity**						
	**Boys**					
**BMI**						
Sleep time	1062	0.43 (−0.08; 0.93)	0.100	330	−**0.93 (**−**1.80;** −**0.07)**	**0.035**
Skip breakfast	1091	**1.27 (0.79; 1.75)**	**<0.001**	449	**1.11 (0.35; 1.87)**	**0.004**
Sedentary behaviour	1091	0.29 (−0.26; 0.83)	0.302	449	−0.73 (−2.39; 0.93)	0.392
Physical activity	1091	−0.43 (−1.08; 0.22)	0.195	449	−0.05 (−0.83; 0.74)	0.910
**Waist circumference**						
Sleep time	1042	0.55(−0.65; 1.74)	0.369	330	−2.17(−4.43; 0.09)	0.060
Skip breakfast	1071	**2.61 (1.47; 3.74)**	**<0.001**	449	**2.13 (0.12; 4.15)**	**0.038**
Sedentary behaviour	1071	0.60 (−0.68; 1.88)	0.358	449	−0.73 (−5.09; 3.63)	0.744
Physical activity	1071	−0.63 (−2.16; 0.90)	0.418	449	−0.77 (−2.87; 1.33)	0.472
**Waist to heigh**t						
Sleep time				330	−0.01 (−0.02; 0.00)	0.091
Skip breakfast	1071	**0.02 (0.01; 0.03)**	**<0.001**	449	**0.02 (0.01; 0.03)**	**0.005**
Sedentary behaviour	1071	0.00 (−0.00; 0.01)	0.320	449	−0.01 (−0.03; 0.02)	0.71
Physical activity	1071	−0.01 (−0.01; 0.00)	0.270			

Adjusted by potential confounders: center (only in HELENA-CSS), age and education mother. Significant associations are in bold.

EBRBs: energy balance-related behaviours, BMI: body mass index, HELENA-CSS: Healthy Lifestyle in Europe by Nutrition in Adolescence cross-sectional study. BRACAH study: Brazilian Cardiovascular Adolescent Health study, 95%CI: confidence interval, coef β: beta coefficient.

### Joint associations sleep time & EBRBs on adiposity

Tables 3 and 4 show the influence of sleeping time on the association between the studied EBRBs and the body composition outcomes. European girls even sleeping adequately (≥8 h/day) experienced an increase on the indicators of total and abdominal obesity when they skipped breakfast and had a higher SB (>2 h/day). Concerning the duration of sleeping on European boys, both for adequate (≥8 h/day) and inadequate duration (<8 h/day), the positive association with obesity indicators happened when adolescents skipped breakfast. Likewise, in Brazilian boys when they skipped breakfast, there was an increase in average of 1.69 kg/m^2^ in BMI and 0.02 in WHtR even when they slept properly. Regarding the risk for obesity this behaviour of skipping breakfast apparently had an association on this population.

**Table 3:** Adjusted* association between EBRBs and general and abdominal obesity stratified by sleep time among adolescents’ girls studied from HELENA-CSS and BRACAH study

	Sleep time ≥ 8 h/day						Sleep time < 8 h/day					
	n	HELENA	p	n	BRACAH	p	n	HELENA	p	n	BRACAH	p
		coef β (95%CI)			coef β (95%CI)			coef β (95%CI)			coef β (95%CI)	
**BMI**												
Skip breakfast	862	**1.13 (0.64; 1.61)**	**<0.001**	197	0.27 (−0.70; 1.24)	0.580	439	−0.19 (−0.81; 0.44)	0.560	188	0.26 (−0.73; 1.24)	0.605
Sedentary behaviour	862	0.27 (−0.22; 0.77)	0.274				439	0.44 (−0.21; 1.09)	0.182			
Physical activity	862	−0.13 (−0.80; 0.54)	0.702	200	0.10 (−0.99; 1.18)	0.862	439	−0.31 (−1.11; 0.50)	0.459	190	0.24 (−0.78; 1.27)	0.641
**Waist circumference**												
Skip breakfast	851	**1.97 (0.90; 3.03)**	**<0.001**	197	1.96 (−0.92; 4.85)	0.182	428	−1.61 (−2.63; 0.30)	0.120	188	1.73 (−0.99; 4.44)	0.211
Sedentary behaviour	851	**1.20 (0.12; 2.27)**	**0.029**				428	1.22 (−0.31; 2.74)	0.117			
Physical activity	881	0.01 (−1.42; 1.44)	0.989	200	−1.14 (−4.34; 2.05)	0.482	444	−0.19 (−2.05; 1.68)	0.845	190	0.66 (−2.18; 3.50)	0.646
**Waist to heigh**t												
Skip breakfast	851	**0.02 (0.01; 0.02)**	**<0.001**				428	−0.01 (− 0.01; 0.00)	0.235			
Sedentary behaviour	851	**0.01 (0.00; 0.02)**	**0.010**				428	**0.01 (0.00; 0.02)**	**0.041**			
**Physical activity**												

Adjusted by potential confounders: center (only in HELENA-CSS), age and education mother. Significant associations are in bold.

EBRBs: energy balance-related behaviours, BMI: body mass index, HELENA-CSS: Healthy Lifestyle in Europe by Nutrition in Adolescence cross-sectional study, BRACAH study: Brazilian Cardiovascular Adolescent Health study, CI: confidence interval, coef β: beta coefficient.

**Table 4:** Adjusted* association between EBRBs and general and abdominal obesity stratified by sleep time among adolescents’ boys studied from HELENA-CSS and BRACAH study.

	Sleep time ≥ 8 h/Day						Sleep time < 8 h/day					
	n	HELENA	p	n	BRACAH	p	n	HELENA	p	n	BRACAH	p
		coef β (95%CI)			coef β (95%CI)			coef β (95%CI)			coef β (95%CI)	
**BMI**												
Skip breakfast	712	**1.29 (0.70; 1.88)**	**<0.001**	189	**1.69 (0.43; 2.94)**	**0.009**	350	**1.31 (0.47; 2.15)**	**0.002**	141	0.95 (−0.28; 2.17)	0.130
Sedentary behaviour	712	−0.06 (−0.69; 0.57)	0.855	189	0.25 (−2.35; 2.86)	0.849	350	0.86 (−0.20; 1.93)	0.112	141	−1.35 (−4.62; 1.91)	0.416
Physical activity	712	−0.36 (−1.17; 0.45)	0.379	189	−0.66(− 1.96; 0.65)	0.323	350	−0.28 (−1.41; 0.86)	0.635	141	−0.04 (−1.28; 1.19)	0.944
**Waist circumference**												
Skip breakfast	701	**2.83 (1.35; 4.31)**	**<0.001**	189	3.16 (−0.12; 6.45)	0.059	341	**2.36 (0.56; 4.17)**	**0.010**	141	2.43 (−0.84; 5.69)	0.146
Sedentary behaviour	701	−0.08 (−1.66; 1.49)	0.919	189	2.82 (−3.89; 9.54)	0.410	341	1.76 (−0.50; 4.03)	0.127	141	−2.35 (−11.03; 6.33)	0.596
Physical activity	701	−0.16 (−2.16; 1.83)	0.871	189	− 0.90 (−4.21; 2.41)	0.595	341	−1.04 (−3.48; 1.40)	0.404	141	−0.73 (−4.02; 2.55)	0.662
**Waist to height**												
Skip breakfast	701	**0.02 (0.01; 0.03)**	**<0.001**	189	**0.02 (0.00; 0.04)**	**0.020**	341	**0.02 (0.01; 0.03)**	**0.004**	141	0.02 (−0.00; 0.04)	0.056
Sedentary behaviour	701	−0.00 (−0.01; 0.01)	0.892	189	0.01 (−0.03; 0.04)	0.698	341	0.01 (−0.01; 0.02)	0.264	141	−0.02 (−0.06; 0.03)	0.519
Physical activity	701	−0.00 (−0.01; 0.01)	0.949				341	−0.01 (−0.02; 0.01)	0.296			

Adjusted by potential confounders: center (only in HELENA−CSS), age and education mother. Significant associations are in bold.

EBRBs: energy balance-related behaviours, BMI: body mass index, HELENA-CSS: Healthy Lifestyle in Europe by Nutrition in Adolescence cross-sectional study, BRACAH study: Brazilian Cardiovascular Adolescent Health study, CI: confidence interval, coef β: beta coefficient.

## Discussion

In this study, we observed how inappropriate EBRBs in a sample of adolescents from Europe and Brazil were associated with total and abdominal adiposity. Moreover, how sleep time may act on these behaviours leading to obesity. Studies have reported the association between short sleep duration with PA, SB and food intake related to weight gain, compromising healthy development^37^. The present study evaluates how sleep predicts not only these behaviours, as well as influencing how skipping breakfast is associated with obesity, and the literature has not explored this approach among adolescents. The current study indicates that skipping breakfast is associated with adiposity markers in adolescents independent of sleeping time and sex, being the main finding of our study. Conversely, sleep time did not present a direct relationship with favourable measures for obesity.

Regarding sleep duration the association between obesity in children as in adolescents has been previously observed^38^, and may be more stronger among boys than among girls, being probably explained by the difference of pubertal stage between sexes (changes in body composition)^39^. However, obesity may influence pubertal development, suggesting that this relationship can be bidirectional^40^. Some studies tried to identify associations between sleeping duration and caloric intake as well as physical inactivity, but were not successful^38^. Our study found no direct association between short sleep time and adiposity among adolescents studied after adjusting by covariates (Table 2), which illustrate the influence of maternal education level. Thus, this lack of association is in agreement with longitudinal studies in adolescents, unlike those presented in cross-sectional studies when associations can be more easily observed^41^. A cohort study places the importance of adjustment by important confounders as MVPA and depression (associated with sleep abnormalities such as insomnia) to explain or not, the still-discrete association of sleep time with adiposity among adolescents^42^. Another sample of the HELENA study did not show a relationship between sleeping time and obesity even adjusting for MVPA^32^. Moreover, in our study we agree it was not possible to adjust the analysis in either of two samples, to this factor potentially associated with AO, as physical activity, since it is in the causative variable line.

*Sleeping time* does not seem to be the main predictor of obesity in this population; in addition, we do not also evaluate its pattern and quality, which would allow a greater comprehension of its real role. As in early studies, the shorter duration of sleep is associated with a greater total energy expenditure and consequently lower weight, while sleep has a lower energy demand than any other activity^43^.

In accordance with our findings, where Brazilian boys who slept < 8 h/day showed a decrease in total obesity (Table 2), a large sample of adolescents evaluated longitudinally after adjusting for covariates, the effect of short sleep duration did not predict obesity (*WaveII*), but this association occurred with an increased risk due to SB (37%) and presence of depression (84%). The association was observed between depressed adolescents who tended to sleep-less than non-depressed one (Waves I,II)^44^. Nonetheless, adequate sleep may reduce obesity risk by preserving vitality and attenuating fatigue, thus enhancing PA levels.

*Sedentary behaviours* among European girls were also associated with an increase in 1.19 cm of WC. Equally a great Finnish study [Health Behaviour in School-aged Children (HBSC)], where adolescents girls whose EBRBs are characterised by sedentary behaviour, low PA, but not poor sleep had an increased risk for overweight^45^. As showed in a systematic review, different types of SB may have different impacts on health, as screen time and TV viewing can be the worst associated with body composition and cardiometabolic risk factors^46^. Although we did not find associations between *Physical Activity* with obesity, the SB was associated with abdominal adiposity. As the Study of Early Child Care and Youth Development showed, spending more daily hours in SB from infancy to adolescence, 6 years later an increase in all BMI percentiles was observed, being the highest at the 90^th^ percentile. Spending less time in MVPA and sleep did not explain this association, besides other covariates^47^.

Since behaviours are not isolated a combination of health-related behaviours could have a stronger influence on obesity development. In a prospective study (follow-up 200-days) combinations of health-related behaviours were associated with measures of adiposity and cardiometabolic health in children and adolescents^48^. Danish children who had healthy changes on the combinations of these two behaviours (increase in MVPA and sleep time, or reduced SB), improved their MetS score (metabolic syndrome, a cluster of atherogenic abnormalities that compound the risk factors for cardiovascular diseases and type2 diabetes), than children with reduced MVPA and sleep, and increased SB^48^.

Breakfast should provide 25% of the daily recommended energy intake^49^, *Skipping Breakfast* may influence an unbalanced diet and energy intake during the day, which can make adolescents vulnerable to weight gain. Moreover, regular consumption of breakfast is associated with micronutrient intake (better dietary pattern including fruits and vegetables, and lower consumption of soft drinks)^50^. The beneficial influence of breakfast consumption on adiposity markers may even improve for male adolescents with low PA levels^51^. As our results suggest, skipping breakfast among adolescents increased total and abdominal obesity, regardless where they live, sleep duration and sex. Adolescents at 16 years of age who usually skipped breakfast, after 27 years of follow-up, showed a significant increase in the prevalence of MetS in adulthood (predicted by AO and high fasting glucose) despite BMI, have another meal in the day or unhealthy lifestyle habits^52^. In addition, skipping breakfast over time during childhood as well as in adulthood, may lead to damage of cardiometabolic health [larger WC, higher homeostatic model assessment (HOMA score) and BMI] besides harmful behaviors^53^.

Children have different reasons for skipping breakfast than adults, and parental influence has great importance. Maternal education, especially on the choice of healthy behaviours among adolescents seems to be an important ally. Previous longitudinal studies revealed that approximately 20% of adolescents skip breakfast each day, and the consumption of better patterns of foods like fruits, vegetables and dairy happens in adolescents with better-educated parents than those with less-educated parents^54^. Adolescents who most consumed breakfast are associated with mothers with a higher schooling and those with a lower family income, but this behaviour’s prevalence tends to decrease with age, which could be explained by the gradual decrease in parental influences^55^. Previously a wide-reaching report of the European branch of the World Health Organisation conducted a health behaviour survey, that involved more than 200,000 schoolchildren showed that 61% of adolescents (13 years old) consumed breakfast (most common among boys and decreased with lower socioeconomic status), fell to 55% among those aged 15^50^.

Normal weight children who skipped breakfast were associated with a larger weight gain over time than adolescents who ate breakfast. Otherwise, overweight children who skipped breakfast were leading to a decrease in BMI over time due to a lower daily energy intake^56^. In a large Australian cohort, children were more likely to skip breakfast when their mother was overweight/obese showing an association between mothers’ skipping breakfast and children’s larger BMI^57^.

### Limitation and strengths

The use of the self-reported questionnaire could be a limitation of our study. However, trying to reduce this measure bias we used the reliability methods to assess the behaviours, we applied reliability subjective measures, which have been shown to have a strong validation performance comparable to objective measures (accelerometers)^58^. Despite worldwide changes and the time of development of our studies, currently published papers with more recent datasets has not shown great changes regarding lifestyles and eating habits. As in the DONALD cohort study where temporal trends regarding eating behaviours have been small over the years among children and adolescents. In addition, the nocturnal fasting period has been increasing with age, which suggests an increase in skipping breakfast^59^. On the other hand, the strengths of our study, despite its cross-sectional design, were to provide a considerable and diverse sample of adolescents with data available equally for all behaviours. In addition, cultural and geographic differences and age range among European and Brazilian adolescents, besides some methodological differences between the studies, placed data to be assessed separately.

Seeking dietary healthy habits avoiding “obesogenic” environments, encouraging and increasing physical activity in groups besides adequating sedentary behaviour to reduce it mostly during leisure time should be considered a better lifestyle behaviour. As an array of several EBRBs are more prevalent than each behaviour separately, a combination of several behaviours should be aimed in order to acquire a healthier lifestyle. Understanding and building up better and good habits early is the basis for a healthier life in the future.

## Conclusion

Skipping breakfast is associated with total and abdominal obesity independent of sleeping time in European boys. European and Brazilian adolescents even having adequate sleep time but skipping breakfast presented higher levels of obesity. Likewise, European girls who had sleep time ≥ 8 h/day plus inadequate sedentary behaviours showed higher abdominal obesity.

## Supplementary information

Supplementary information

## Data Availability

The data was not available in the first step due to confidential ethical issues, in accord with the rules of the ethical committee. In case the article will be accepted, the data will be made available.
