# Supplementary material for: Skipping breakfast is associated with adiposity markers especially when sleep time is adequate in adolescents
Source: Sci Rep. 2019 Apr 23;9:6380. doi: 10.1038/s41598-019-42859-7 (PMC6476875; doi:10.1038/s41598-019-42859-7)
Supplement: Supplementary file 1 — Supplementary information [file 41598_2019_42859_MOESM1_ESM.docx]

**Skipping breakfast is associated with adiposity markers especially when sleep time is adequate in adolescents**

**Elsie C.O. Forkert* ^1^_,_ Augusto Cesar Ferreira De Moraes^1^, Heráclito Barbosa Carvalho^1^, Yannis Manios^4^, Kurt Widhalm^5^, Marcela González-Gross^6,7^, Angel Gutierrez^8^, Anthony Kafatos^9^, Laura Censi^10^, Stefaan De Henauw^11^, Luis A. Moreno^2,3,12^**

**1-YCARE** (**Y**outh/**C**hild c**A**rdiovascular **R**isk and **E**vironmental) **Research Group**, Departamento de Medicina Preventiva, Faculdade de Medicina FMUSP, Universidade de Sao Paulo, Sao Paulo, SP, BR; **2- GENUD** (**G**rowth, **E**xercise, **NU**trition and **D**evelopment) **Research Group,** Facultad de Ciencias de la Salud de la Universidad de Zaragoza, , Instituto Agroalimentário de Aragón (IA2), Zaragoza, Spain; **3**-Department of Preventive Medicine - Visiting Professor, School of Medicine, University of São Paulo, São Paulo, Brazil; **4-**Department of Nutrition and Dietetics, Harokopio University, Athens,Greece; **5-**Department of Pediatrics, Paracelsus Medical University, Salzburg, Austria; **6-**ImFINE Research Group. Department of Health and Human Performance**,** Faculty of Physical Activity and Sport-INEF, Technical University of Madrid, and Centro de Investigación Biomédica en Red Fisiopatología de la Obesidad y Nutrición(CIBEROBN), Madrid, Spain; **7-**Institut für Ernährungs-und Lebensmittelwissenschaften–Humanernährung, Rheinische Friedrich-Wilhelms, Universität Bonn, Bonn,Germany; **8-**Department of Medical Physiology, School of Medicine, University of Granada, Granada, Spain; **9-**Preventive Medicine and Nutrition Unit, University of Crete School of Medicine, Heraklion, Crete, Greece; **10-**Council for Agricultural Research and Economics-Research Center for Food and Nutrition,Rome,Italy; **11-**Department of Public Health, Ghent University, Ghent, Belgium; **12-**Insttituto Agroalimentario de Aragón (IA2), Instituto Investigación Sanitaria Aragón (IIS Aragón) and Centro de Investigación Biomédica en Red Fisiopatología de la Obesidad y Nutrición (CIBEROBN), Zaragoza, Spain.


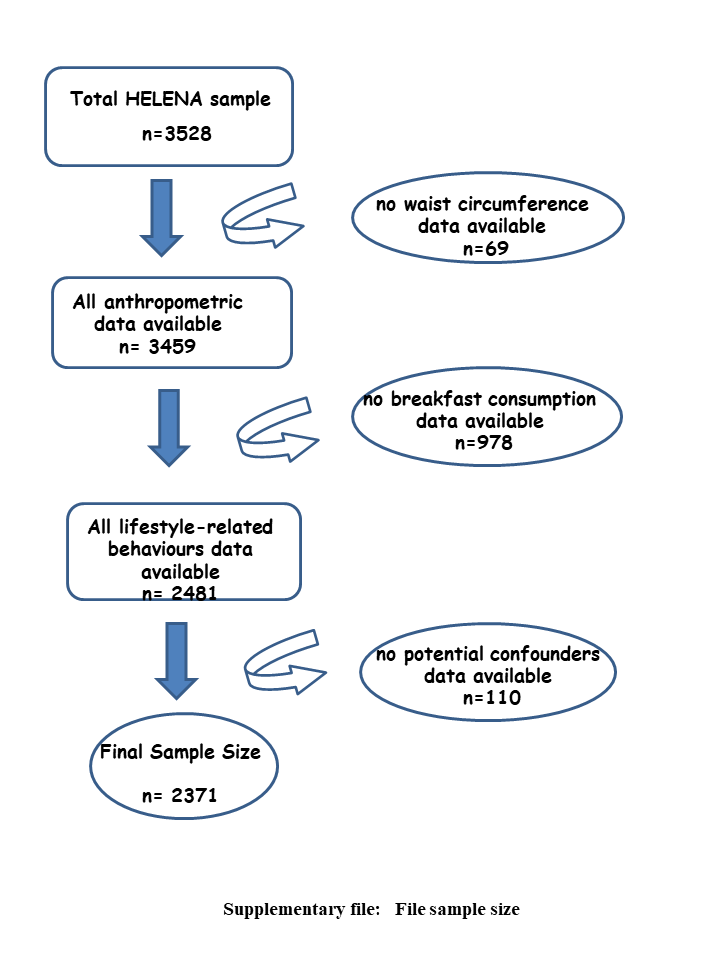


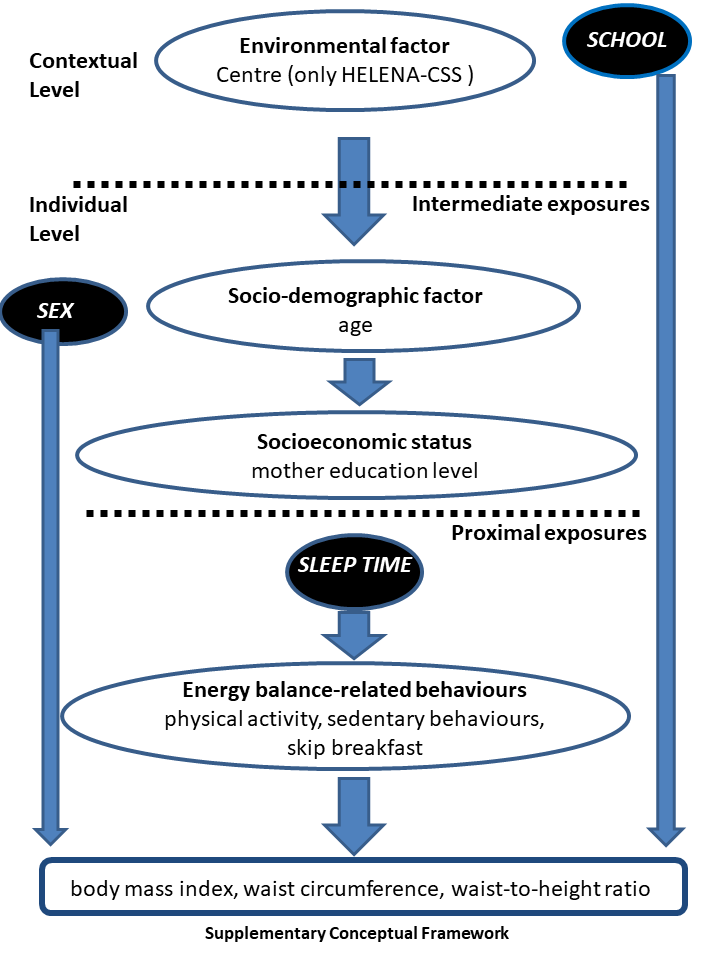


| **Table S1. Unadjusted association between EBRBs with adiposity indicators, among adolescents from HELENA-CSS and BRACAH study** | | | | | | | | | |
| --- | --- | --- | --- | --- | --- | --- | --- | --- | --- |
| **GIRLS** | | | | |  | **BOYS** | | | |
| **EBRBs** |  | **BMI** | | | | | | | |
|  |  | **HELENA** |  | **BRACAH** |  |  | **HELENA** |  | **BRACAH** |
|  | **n** | **coef β (95%CI)** | **n** | **coef β (95%CI)** |  | **n** | **coef β (95%CI)** | **n** | **coef β (95%CI)** |
| **Sleep time** | 1350 | **(p=0.02)** | 390 | (p=0.883) |  | 1121 | **(p<0.001)** | 331 | **(p=0.033)** |
| **≥8h/day** |  | Ref. |  | Ref. |  |  | Ref. |  | Ref. |
| **<8h/day** |  | **0.47 (0.07;0.87)** |  | -0.05(-0.72 ; 0.62) |  |  | **0.93(0.44 ;1.42)** |  | **-0.93(-1.79 ; -0.08)** |
| **Skip breakfast** | 1372 | **(p<0.001)** | 540 | (p=0.649) |  | 1154 | **(p<0.001)** | 451 | **(p=0.002)** |
| **no** |  | Ref. |  | Ref. |  |  | Ref. |  | Ref. |
| **yes** |  | **0.88(0.50 ; 1.26)** |  | -0.14( -0.73 ; 0.46) |  |  | **1.60(1.13 ; 2.06)** |  | 1.17( 0.41 ; 1.92) |
| **Sedentary behaviour** | 1372 | **(p=0.015)** | 540 | (p=0.759) |  | 1154 | (p=0.061) | 451 | (p=0.432) |
| **≤2h/day** |  | Ref. |  | Ref. |  |  | Ref. |  | Ref. |
| **>2h/day** |  | **0.47 (0.09 ; 0.85)** |  | 0.27(-1.45 ; 1.98) |  |  | 0.53 (-0.03 ; 1.08) |  | -0.65(-2.27 ; 0.97) |
| **Physical activity** | 1372 | (p= 0.80) | 540 | (p=0.225) |  | 1154 | (p= 0.674) | 451 | (p=0.805) |
| **≥60min/day** |  | Ref. |  | Ref. |  |  | Ref. |  | Ref. |
| **<60min/day** |  | 0.06(-0.45 ; 0.58) |  | 0.40(-0.25 ; 1.04) |  |  | -0.14(-0.78 ; 0.51) |  | -0.10(-0.89 ; 0.69) |
| **EBRBs** |  | **Waist Circumference** | | | | | | | |
|  |  | **HELENA** |  | **BRACAH** |  |  | **HELENA** |  | **BRACAH** |
|  | **n** | **coef β (95%CI)** | **n** | **coef β (95%CI)** |  | **n** | **coef β (95%CI)** | **n** | **coef β (95%CI)** |
| **Sleep time** | 1325 | (p=0.250) | 390 | (p=0.463) |  | 1101 | **(p=0.002)** | 331 | **(p=0.042)** |
| **≥8h/day** |  | Ref. |  | Ref. |  |  | Ref. |  | Ref. |
| **<8h/day** |  | 0.52(-0.37 ; 1.42) |  | -0.72(-2.65 ; 1.21) |  |  | **1.83(0.66 ; 3.00)** |  | **-2.34(-4.59 ; -0.09)** |
| **Skip breakfast** | 1347 | **(p=0.006)** | 540 | (p=0.417) |  | 1134 | **(p<0.001)** | 451 | **(p=0.026)** |
| **no** |  | Ref. |  | Ref. |  |  | Ref. |  | Ref. |
| **yes** |  | **1.19(0.35 ; 2.04)** |  | 0.67(-0.95 ; 2.29) |  |  | **3.21(2.09 ; 4.33)** |  | 2.28(0.28 ; 4.29) |
| **Sedentary behaviour** | 1347 | **(p=0.002)** | 540 | (p=0.534) |  | 1134 | (p=0.193) | 451 | (p=0.929) |
| **≤2h/day** |  | Ref. |  | Ref. |  |  | Ref. |  | Ref. |
| **>2h/day** |  | **1.34(0.49 ; 2.19)** |  | 1.49(-3.20 ; 6.18) |  |  | 0.87(-0.44 ; 2.18) |  | -0.20(-4.49 ; 4.10) |
| **Physical activity** | 1347 | (p=0.51) | 540 | (p=0.929) |  | 1134 | (p=0.784) | 451 | (p=0.368) |
| **≥60min/day** |  | Ref. |  | Ref. |  |  | Ref. |  | Ref. |
| **<60min/day** |  | 0.39 (-0.75 ; 1.52) |  | 0.08(-1.68 ; 1.84) |  |  | 0.21 (-1.32 ; 1.75) |  | -0.98(-3.11 ; 1.15) |
| **EBRBs** |  | **Waist to Height** | | | | | | | |
|  |  | **HELENA** |  | **BRACAH** |  |  | **HELENA** |  | **BRACAH** |
|  | **n** | **coef β (95%CI)** |  | **coef β (95%CI)** |  | **n** | **coef β (95%CI)** | **n** | **coef β (95%CI)** |
| **Sleep time** | 1325 | (p=0.21) | 390 | (p=0.759) |  | 1101 | (p=0.559) | 331 | **(p=0.068)** |
| **≥8h/day** |  | Ref. |  | Ref. |  |  | Ref. |  | Ref. |
| **<8h/day** |  | 0.00 (-0.00 ; 0.01) |  | -0.00(-0.01 ; 0.01) |  |  | 0.00 (-0.01 ; 0.01) |  | **-0.01(-0.02 ; 0.00)** |
| **Skip breakfast** | 1347 | **(p<0.001)** | 540 | (p=0.850) |  | 1134 | **(p<0.001)** | 451 | **(p=0.003)** |
| **no** |  | Ref. |  | Ref. |  |  | Ref. |  | Ref. |
| **yes** |  | **0.01(0.01 ; 0.02)** |  | 0.00(-0.01 ; 0.01) |  |  | **0.02 (0.01 ; 0.02)** |  | **0.02(0.01 ; 0.03)** |
| **Sedentary behaviour** | 1347 | **(p<0.001)** | 540 | (p=0.317) |  | 1134 | **(p=0.087)** | 451 | (p=0.818) |
| **≤2h/day** |  | Ref. |  | Ref. |  |  | Ref. |  | Ref. |
| **>2h/day** |  | **0.01(0.01; 0.02)** |  | 0.02(-0.01 ; 0.04) |  |  | **0.01(-0.00; 0.01)** |  | -0.00(-0.03 ; 0.02) |
| **Physical activity** | 1347 | (p=0.696) | 540 | (p=0.311) |  | 1134 | (p=0.252) | 451 | (p=0.863) |
| **≥60min/day** |  | Ref. |  | Ref. |  |  | Ref. |  | Ref. |
| **<60min/day** |  | 0.00(-0.01 ; 0.02) |  | 0.01(-0.01 ; 0.02) |  |  | -0.01(-0.01 ; 0.00) |  | -0.00(-0.01 ; 0.01) |
| **Significant associations are in bold. EBRBs: energy balance-related behaviours, BMI: body mass index, HELENA-CSS: Healthy Lifestyle in Europe by Nutrition in Adolescence cross-sectional study, BRACAH study: Brazilian Cardiovascular Adolescent Health study, 95%CI: confidence interval, coef β: beta coefficient.** | | | | | | | | | |
